# From vision correction to health promotion: a public health approach to myopia

**DOI:** 10.3389/fpubh.2026.1734857

**Published:** 2026-03-03

**Authors:** Naveen Kumar Challa

**Affiliations:** Department of Optometry, College of Applied Medical Sciences, Qassim University, Buraidah, Saudi Arabia

**Keywords:** clinical practice, health promotion, myopia, public health, quality of life

## 1 Introduction

Myopia, or nearsightedness, is rising at an alarming rate and is rapidly becoming a global public health concern. By 2050, nearly half of the world's population is expected to be myopic (1, 2). While glasses, contact lenses, and laser surgery have long served as effective methods for vision correction, the unprecedented rise in myopia demands a more comprehensive and patient-centered response one that not only addresses refractive error but also considers lifestyle, emotional wellbeing, and long-term eye health.

## 2 Beyond vision correction: the evolution of myopia management

In recent years, myopia management has evolved well-beyond the goal of simply improving visual acuity. Treatments such as orthokeratology (Ortho-K) lenses, low-dose atropine eye drops, and peripheral defocus contact lenses have shown significant promise in slowing myopia progression (3–9). However, these interventions are not standalone solutions. Their effectiveness is influenced by several factors, including patient consistency, timely access to treatment, and personalized care plans (10). It is not just a matter of having the right tools, it is about ensuring they are used in ways that align with the patient's daily life and long-term needs.

## 3 Lifestyle factors and modern challenges

Lifestyle also plays a critical role in myopia development and progression. Research indicates that children who spend more time outdoors and limit screen exposure tend to have a lower risk of worsening myopia (11). While this advice may appear straightforward, the reality is more complex. In today's digitally driven world, screens are integrated into nearly every aspect of a child's life from education and communication to entertainment. Encouraging outdoor activity and setting boundaries around screen time now require deliberate, coordinated efforts by parents, teachers, and healthcare providers alike.

## 4 Emotional and psychological dimensions of myopia

The impact of myopia extends beyond the physical challenges of blurred vision. For many children and adolescents, it also brings emotional and psychological burdens (12, 13). Difficulty seeing the board in class, squinting during daily tasks, or feelings of self-consciousness from wearing glasses can negatively affect self-confidence and peer interactions. A truly patient-first approach must go beyond the clinical setting to include emotional support, open dialogue, and accessible education about eye health. When families understand the nature of myopia and are supported without fear or confusion, they are more empowered to make informed decisions.

## 5 The expanding role of eye care providers

Eye care providers play a pivotal role in this evolving model of care. They must embrace a broader view, one that includes not only flexible and individualized treatment plans but also active patient education and the thoughtful integration of technology. Digital health tools, including telemedicine and mobile apps, can facilitate ongoing engagement by making it easier for patients to track vision changes, attend follow-up appointments, and communicate with providers (14). These innovations can enhance both accessibility and adherence, particularly in younger or busy populations.

## 6 Bridging the gap: equity and access

Nevertheless, equity in access remains a critical challenge. Many families face significant barriers such as financial constraints or geographic isolation, which limit their ability to receive timely and specialized eye care (15). This gap in access is a major concern that requires collaborative solutions. Policymakers must work to expand public vision care initiatives, and industry stakeholders should prioritize the development of more affordable and scalable treatments. Local solutions such as school-based screenings, community outreach programs, and subsidized services can also help bridge these disparities and ensure early intervention.

## 7 Toward a patient-centered, preventive future

Ultimately, managing myopia is not solely about reaching 20/20 vision. It is about enhancing quality of life, preserving lifelong eye health, and creating a care model that is as compassionate as it is effective. A modern, human-centered approach to myopia management must blend scientific innovation with practical accessibility, and clinical expertise with emotional understanding.

To address the growing myopia epidemic, we need to move from a reactive model focused only on correction, to a proactive, preventive framework rooted in personalized care. This means integrating medical treatment with lifestyle modification, behavioral change, and technological support. It also requires looking beyond the individual to the broader healthcare systems, education, policy that shape the environment in which patients live.

## 8 Conclusion: seeing beyond the prescription

As we look toward the future, our goal should be clear: not just better vision, but better lives. Myopia management must evolve into a compassionate, inclusive, and sustainable endeavor one that sees the patient not merely through the lens of their prescription, but as a whole person.
